# Generation of an Rx-tTA: TetOp-Cre Knock-In Mouse Line for Doxycycline Regulated Cre Activity in the Rx Expression Domain

**DOI:** 10.1371/journal.pone.0050426

**Published:** 2012-11-27

**Authors:** Timothy F. Plageman, Richard A. Lang

**Affiliations:** 1 Visual Systems Group, Children's Hospital Research Foundation, Cincinnati Children’s Hospital Medical Center, Cincinnati, Ohio, United States of America; 2 Division of Pediatric Ophthalmology, Children's Hospital Research Foundation, Cincinnati Children’s Hospital Medical Center, Cincinnati, Ohio, United States of America; 3 Division of Developmental Biology, Children's Hospital Research Foundation, Cincinnati Children’s Hospital Medical Center, Cincinnati, Ohio, United States of America; 4 Department of Ophthalmology, University of Cincinnati, Cincinnati, Ohio, United States of America; Instituto Gulbenkian de Ciência, Portugal

## Abstract

Genetic deletion of mouse genes has played a crucial role in our understanding of embryonic eye development. Transgenic, tissue specific Cre recombinase expression in various eye structures has facilitated these experiments. However, an early expressing, temporally-regulated, optic vesicle-specific Cre line has not been available. In this report, we detail the generation and analysis of a knock-in, inducible Cre line designed to drive recombination specifically within the Rx expression domain. Crossing this line with a reporter line demonstrates that recombination can be mediated within the early optic vesicle and throughout retinal development. Recombination can also be mediated in the Rx-expressing, ventral diencephalon and future posterior pituitary gland. Furthermore, it was demonstrated that dietary doxycycline could effectively modulate Cre activity. This line has the potential to facilitate conditional knock-out experimentation to study early retina and/or posterior pituitary development.

## Introduction

The field of developmental biology has greatly benefitted from the use of an evolving set of genetic tools. Utilizing conditional, recombination-based strategies, mouse genes can be deleted in a specific cell type and in some instances with temporal control. This is accomplished through the use of mouse lines that express the bacteriophage gene Cre-recombinase (Cre) from cell type-specific promoters [1]. When crossed with an engineered mouse line possessing loxP sites flanking a targeted region of a gene of interest, Cre deletes between the loxP sites and creates a somatic mutation [2]. In addition to spatial control, Cre expression can also be regulated temporally [3]. Rather than directly regulating the Cre gene, the tissue specific promoter can be placed upstream of a transcriptional activator whose activity is affected by the presence or absence of diet supplemented drugs. In tetracycline-inducible systems, genetic deletion will occur only after the mouse is dosed with tetracycline. Because these methods are dependent on tissue-specific and/or inducible Cre expression, the generation of novel Cre-expressing mouse lines with particular embryonic tissue specificity is extremely valuable to developmental biologists.

**Figure 1:**
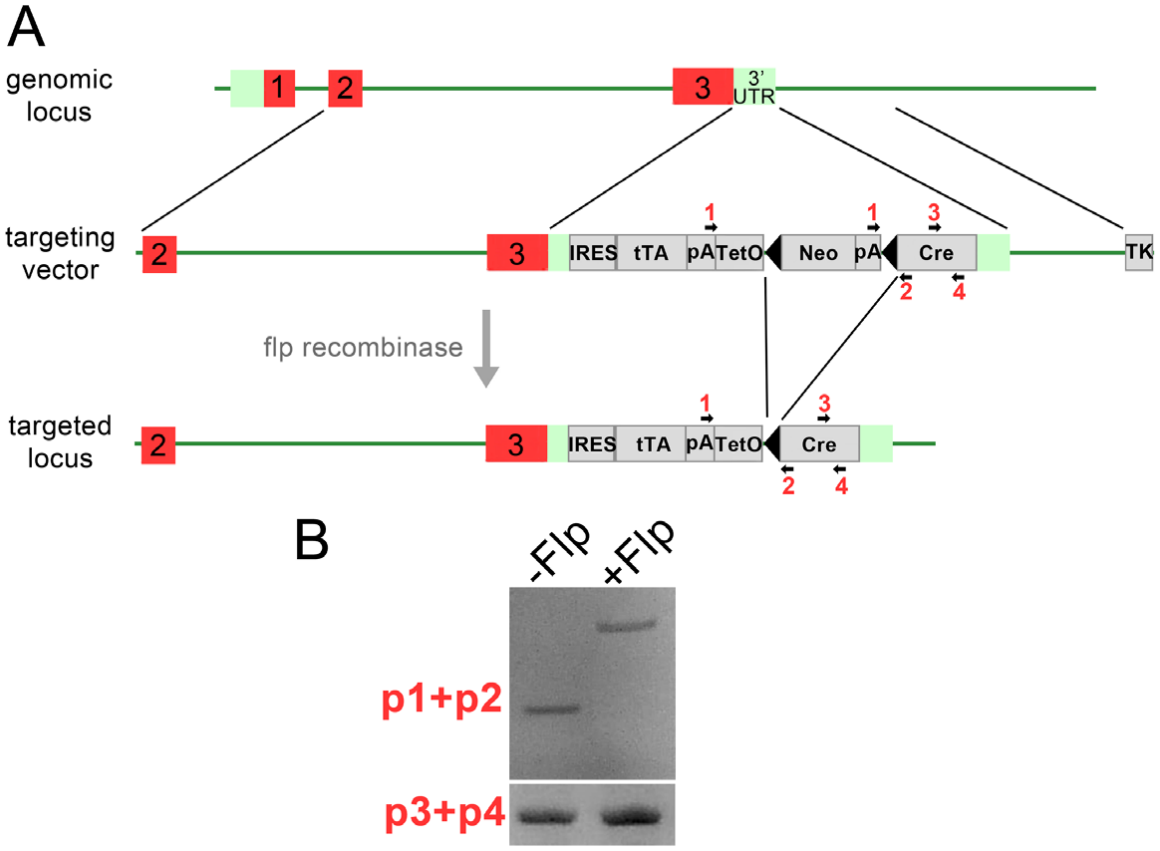
Diagram of the targeting strategy to generate the Rx-tTA: TetOp-Cre knock-in allele. A. The targeting vector consists of homology arms to exon 2, intron2/3, exon 3, 3′ UTR and 3′ genomic sequence. Sequence containing the tTa/Cre cassette and the neomycin resistance gene was inserted within the 3′UTR. The final targeted locus following the action of flp recombinase is also depicted. The black arrowheads indicate FRT sites and the red numbers denote the location of primers used for genotyping. B. PCR was performed with the indicated primers on genomic DNA from Rx-tTA: TetOp-Cre mice crossed with the flp recombinase line. IRES: internal ribosomal entry site, tTA: tetracycline transactivator coding sequence, TetO: operator of the *E. coli* tetracycline resistance gene, Neo: neomycin resistance gene, Cre: Cre-recombinase coding sequence, pA: poly-adenylation sequence, TK: Thymidine kinase.

The study of early mouse eye development has been aided by several Cre-recombinase transgenes. For example, much has been learned from lens induction and morphogenesis of the lens pit using the Le-Cre line, which utilizes portions of a *Pax6* enhancer and promoter elements to drive Cre expression throughout lens development [4]. The advantage of this line has been the strong expression of Cre spanning the onset of lens development in the placode to the mature adult lens. An analogous Cre transgene would be helpful to study early development of the optic vesicle, the retinal precursor. Several lines express Cre in the early retina [5]–[7], however, most of them are not broadly expressed throughout the optic vesicle earlier than E10.5, a time point when many events of retinal development have already occurred. One exception to this is the Six3-Cre line [8]. This line drives Cre expression beginning at E9.0 but is not broadly expressed throughout the much of the optic vesicle until E9.5 [8], [9]. Another exception is a line utilizing the Medaka *rx3* regulatory elements that drives Cre expression at the onset of retinal formation [10]. While useful in several studies [9]–[13], it was noted that this line occasionally expresses Cre in the lens, heart, neural crest, and olfactory placode [10], [12]. Occasional expression in the lens can be particularly disruptive for experiments designed to selectively analyze the contribution of genes in the retina that also play a significant role during lens development. In addition, both the Six3-Cre and Rx-Cre transgenic lines have the disadvantage of expressing Cre throughout development, potentially preventing functional analysis of some genes at a later time-point. For these reasons we sought to develop a Cre line that has the capability to robustly recombine floxed alleles within the entire early optic vesicle and throughout retinal development but can also be regulated in an inducible manner.

**Figure 2:**
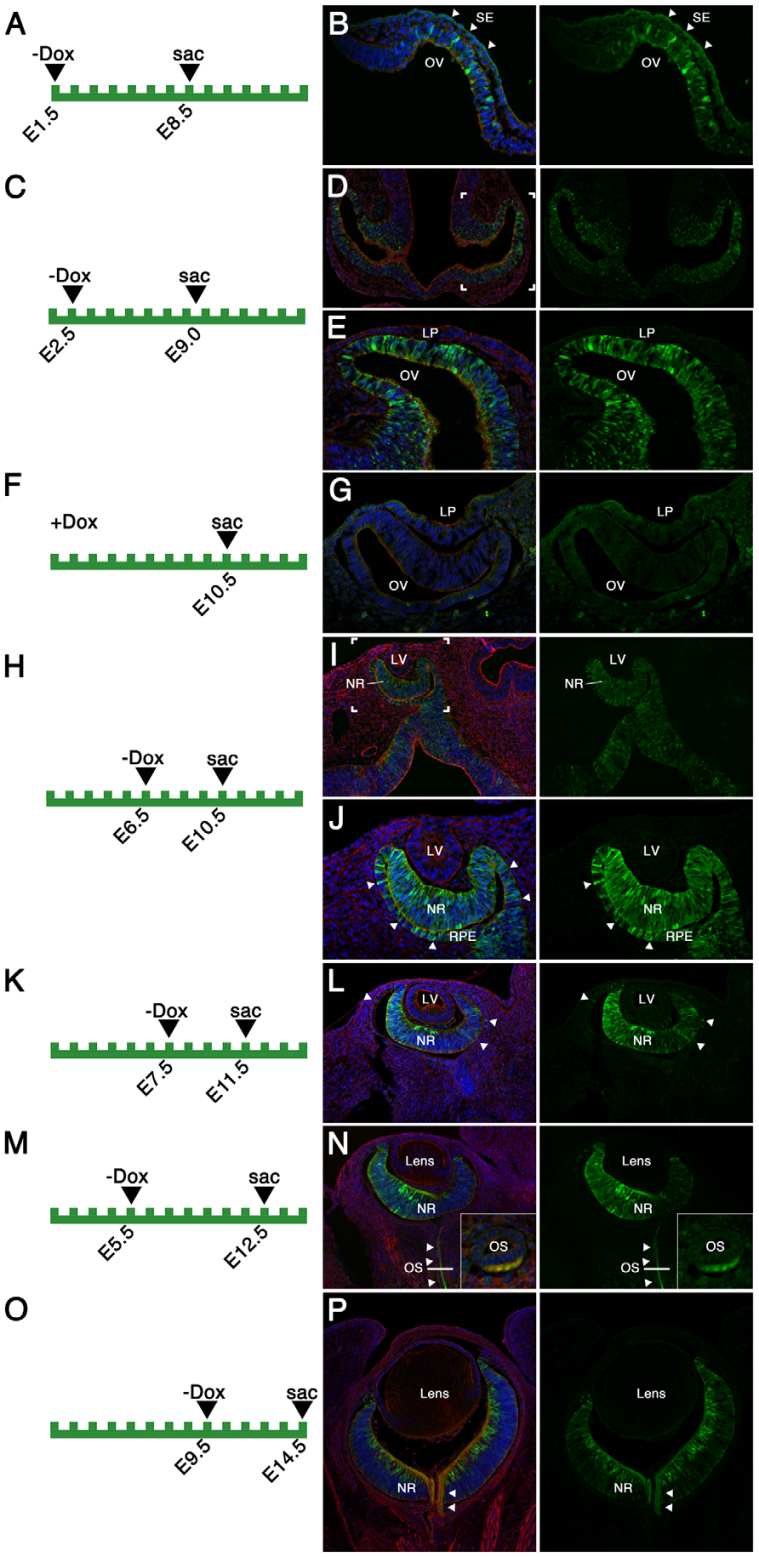
Analysis of EGFP reporter expression of Rx-tTA: TetOp-Cre; Z/EG doubly heterozygous mice during embryonic eye development. (A–P) The time-points of dietary doxycycline withdrawal (−Dox) and embryo dissection (sac) are depicted on the diagrams in the left panels. For panel F, +Dox indicates that doxycycline was not withdrawn. Coronal cryosections (dorsal side oriented to the left) of Rx-tTA: TetOp-Cre; Z/EG doubly heterozygous mice were stained with phalloidin (red), and Hoeschst (blue). Green fluorescence indicates the expression of EGFP under the control of Cre-recombinase. Arrowheads in panel B indicate the lack of EGFP expression in the surface ectoderm (SE) in contrast to the EGFP positive cells in the optic vesicle (OV). The bracketed region in panels D and I is shown under a higher magnifcation in panels E and J, respectively. Arrowheads in panels J and L indicate EGFP positive cells in the retinal pigmented epithelium (RPE). Arrowheads in panel N and P indicate positive staining of the retinal ganglion cells projecting from the neural retina. The inset panel in N displays a sagittal section through the optic stalk (OS) at approximately the position indicated by the solid white line. LV: lens vesicle; NR, neural retina.

## Results and Discussion

Rx is a transcription factor initially expressed approximately at E8.0 in patches of the anterior neural plate that will eventually invaginate to form the optic vesicles [14]. Expression of Rx persists during much of retinal development and is also observed in the ventral forebrain of early mouse embryos [14]. This expression pattern and the previous success of the above-mentioned Rx-Cre line prompted the use of this genetic locus to generate an inducible, retinally expressing Cre line. To do this, a targeting vector containing the tTA expression cassette was designed with complementary sequence to the final mouse Rx exon within the 3′ untranslated region (Fig. 1A). The tTA regulator is the key component of the TetOff system and is designed to prevent Cre expression in the presence of tetracycline/doxycycline [3]. Upon removal of tetracycline/doxycycline, Rx-dependent expression of the tTA fusion transcription factor can bind to the Tet operator and drive Cre expression. Confirmation of correct targeting was confirmed with PCR assays in different ES cell clones (data not shown). Several chimeras were generated from mouse blastocysts injected with targeted ES cells, and of these one produced progeny that inherited the Rx-tTA: TetOp-Cre knock-in allele. The neo cassette was subsequently removed from the founder line by crossing with Flp recombinase expressing mice and its removal was confirmed by PCR (Fig. 1B).

**Figure 3:**
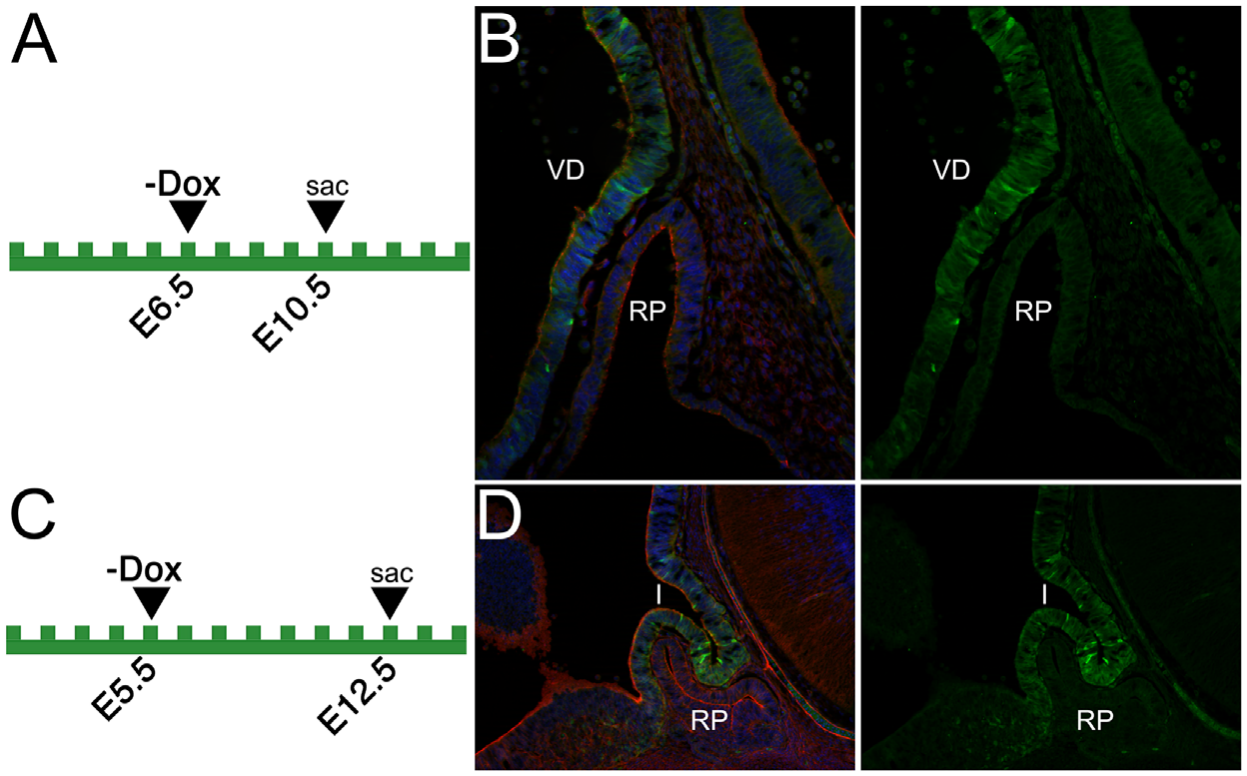
Analysis of EGFP reporter expression of Rx-tTA: TetOp-Cre; Z/EG doubly heterozygous mice during early pituitary development. (A–D) The time-points of dietary doxycycline withdrawal (−Dox) and embryo dissection (sac) are depicted on the diagrams in the left panels. Sagittal cryosections of Rx-tTA: TetOp-Cre; Z/EG doubly heterozygous mice were stained with phalloidin (red), and Hoeschst (blue). Green fluorescence indicates the expression of EGFP under the control of Cre-recombinase. VD; ventral diencephalon, RP; Rathke’s pouch, I; infundibulum.

To determine whether the Rx-tTA: TetOp-Cre allele drives Cre expression in the developing retina, Rx-tTA: TetOp-Cre transgenic mice were crossed with the Z/EG reporter line that expresses EGFP in cells where recombination occurs [15]. Prior to mating, female mice were maintained on doxycycline and withdrawn at different points during gestation. In an initial experiment, doxycycline was withdrawn from E1.5 and double heterozygous embryos analyzed at E8.5 (Fig. 2A). This resulted in a few positive cells throughout the optic vesicle (Fig. 2B). The surrounding mesenchymal and surface ectoderm cells (arrowheads) were negative for reporter activity (Fig. 2B). With doxycycline withdrawal at E2.5, strong reporter activity was observed throughout the optic vesicle and future optic stalk at E9.0 (Fig. 2C–E). In a control experiment, doxycycline was not removed from the diet and embryos derived from this cross were analyzed at E10.5. Reporter activity was not observed anywhere in the embryo including the eye (Fig. 2F–G) indicating that the Rx-tTA: TetOp-Cre responds well to dietary doxycycline and can function in an inducible fashion. Expression of the reporter in double heterozygotes was also analyzed at E10.5 after doxycycline withdrawal at E6.5 (Fig. 2H–J), at E11.5 after doxycycline withdrawal at E7.5 (Fig. 2K–L), at E12.5 after doxycycline withdrawal at E5.5 (Fig. 2M–N), and at E14.5 after doxycycline withdrawal at E9.5 (Fig. 2O–P). At all of these stages, reporter expression is observed throughout the neural retina but never observed in the lens or ocular mesenchyme. Some variability in the expression levels at E11.5 and E12.5 was also observed where the dorsal neural retina exhibits stronger reporter activity than the ventral retina (Fig. 2L, N). In addition, reporter activity is present in some cells of the retinal pigmented epithelium (RPE) with some Dox withdrawal timings in a variable fashion (arrowheads in Fig. 2J, L) and in the retinal ganglion cell axons tracking along the ventral optic stalk (arrowheads in Fig. 2N, P; sagittal section in 2N). Reporter activity could also be observed in the neural retina after only 2 days of doxycycline withdrawal (E8.5–E10.5), however, expression was patchy and completely absent from the RPE (data not shown).

**Figure 4:**
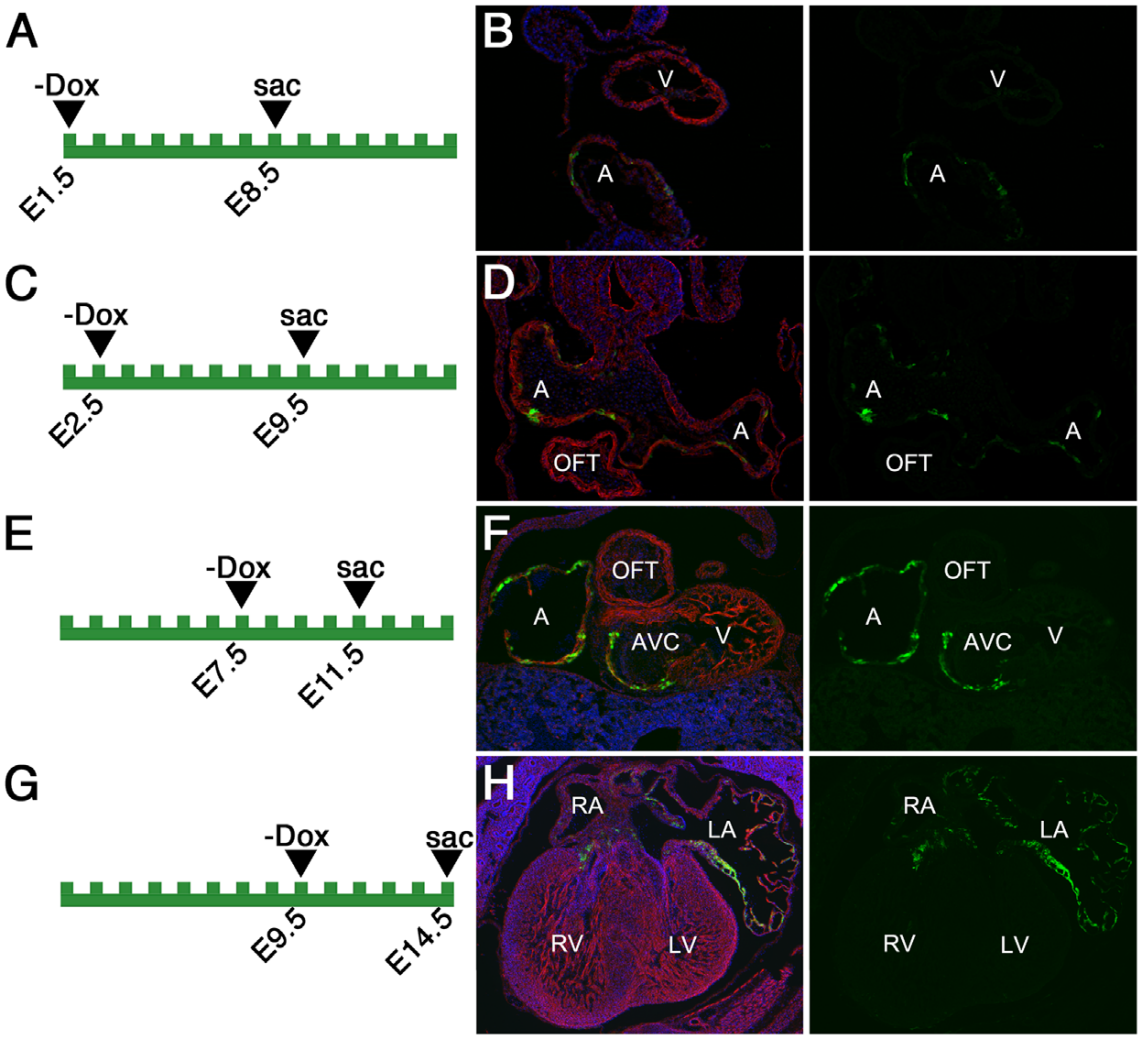
Analysis of EGFP reporter expression of Rx-tTA: TetOp-Cre; Z/EG doubly heterozygous mice during early heart development. (A–H) The time-points of dietary doxycycline withdrawal (−Dox) and embryo dissection (sac) are depicted on the diagrams in the left panels. Coronal (B,D,F) or transverse (H) cryosections of Rx-tTA: TetOp-Cre; Z/EG doubly heterozygous mice were stained with phalloidin (red), and Hoeschst (blue). Green fluorescence indicates the expression of EGFP under the control of Cre-recombinase. A: atria, V: ventricle; OFT: outflow tract; AVC: atrioventricular canal; RA: right atrium; LA: left atrium; RV: right ventricle; LV: left ventricle.

The Rx gene is expressed in the ventral diencephalon in early embryonic development and is required for normal development of the posterior pituitary [14], [16]. To determine if Cre activity is found in the developing pituitary of Rx-tTA: TetOp-Cre; Z/EG embryos, reporter activity was analyzed in sagittal sections (Fig. 3). At E10.5 after E6.5 Dox withdrawal, Cre reporter activity was observed throughout the ventral diencephalon (Fig. 3A–B), consistent with the reported expression pattern of Rx. Cre activity remains strong at E12.5 in the infundibulum, the posterior pituitary precursor, as it invaginates concomitantly with Rathke’s pouch (Fig. 3C–D). Because of the strong Cre activity observed, this line could potentially be useful for studying posterior pituitary development.

In addition to the eye and posterior pituitary, expression of Cre was also consistently observed in the developing atrial myocardium of the heart (Fig. 4). At E8.5, cells in the posterior heart tube myocardium, a region fated to form the atrial chambers, are positive for reporter expression. Although a large number of cells are positive, it is clear that a significant number are negative for reporter activity (Fig. 4A–B). The spotty reporter activity in the atrial myocardium is consistently observed at E9.5 (Fig. 4C–D), E11.5 (Fig. 4E–F), and E14.5 (Fig. 4G–H). The reporter activity is not uniform in the atria but is never found in the ventricular or outflow tract myocardium, or in the endothelial cells lining the cardiac chambers. Because of the patchy nature of Cre activity in the atrium, the Rx-tTA: TetOp-Cre knock-in allele may not serve as an optimal atrial myocardium driver. It is unclear why atrial expression was observed, as Rx expression has not been reported in the heart. However, it is consistent with the report of occasional cardiac expression in the previously generated Rx-Cre line [10].

Although none of the Rx-tTA: TetOp-Cre knock-in positive progeny fed with a constant diet of doxycycline (n = 14) had any observable phenotype, we noticed that when doxycycline is never fed to pregnant dams the pups were runted, lacked all observable eye structures and resembled the *Rx* knock-out mice (n = 3). Because a doxycycline diet prevents embryonic defects under several different doxycycline regimens and embryonic stages (Figs 2, 3, 4), we do not believe that the *Rx* gene was disrupted from the targeting. Instead, it is more likely that the phenotype is due to Cre-mediated toxicity, a phenomena documented in other Cre expressing mice [17], [18]. If the early *Rx*-expressing cells, which are retinal precursors, are dying it may explain the phenotypic similarity to the *Rx* null mice. It is also important to note that we observed a small eye phenotype in Rx-tTA: TetOp-Cre knock-in embryos when the lag time between embryo analysis and doxycycline withdrawal is too long. For example, when doxycycline withdrawal is performed at E4 and the embryos analyzed at E11.5 the Rx-tTA: TetOp-Cre positive embryos had smaller eyes than their wild-type littermates (data not shown). With these data, we recommend minimizing the period of doxycycline withdrawal that permits analysis at a given stage.

The Rx-tTA: TetOp-Cre knock-in mouse line was designed to enable the analysis of specific genes in the developing retina from the onset of its development. This line may be especially useful for genetically ablating genes required for retinal induction and/or early morphogenesis. This report also demonstrates the utility of this line to carry out genetic experiments in the posterior pituitary as well. It is important to point out that the Cre activity in the heart might be a complicating factor as genes required for heart development might disrupt cardiac function and cause embryonic death precluding analysis of the eye or pituitary at desired stages. Fortunately the inducible nature of this line could limit this effect.

## Methods

### Ethics Statement

All experiments were performed in accordance with institutional guidelines under Institutional Animal Care and Use Committee (IACUC) approval at Cincinnati Children's Hospital Research Foundation (CCHRF). IACUC at CCHRF approved the study described in this manuscript with Animal Use Protocol number 0B12097.

### Gene Targeting

Design, construction of the Rx-tTA: TetOp-Cre targeting vector, and the subsequent steps to generate Rx-tTA: TetOp-Cre heterozygous mice were performed by the Gene Targeted Mouse Service Core at the University of Cincinnati. In short, the vector was designed in order to insert a 5.0 kb cassette containing IRES-tTA and Cre recombinase driven by the TetO promoter in the 3′-UTR (104 bp from the stop codon) of the Rx gene. The cassette was flanked by 3.4 kb and 2.7 kb of homologous arms, respectively. A neomycin resistance gene and a thymidine kinase gene were included for positive and negative selection. The construct was electroporated into mouse ES cells derived from a 129Sv strain, and ES cell clones with the correctly targeted allele were identified by PCR and confirmed by Southern blot analysis. Correctly targeted ES cell clones were injected into blastocysts to generate chimeras, which were then bred to obtain ES cell-derived offspring, as determined by the presence of agouti coat color. Agouti mice were further analyzed by PCR for the presence of targeted allele. Rx-tTA: TetOp-Cre heterozygous mice were crossed to flp recombinase mice to remove the neo cassette to obtain neo- mice, which were then further bred with wildtype mice to remove the flp transgene (Rodriquez et al., 2000). The Rx-tTA: TetOp-Cre knock-in will be available to the research community upon acceptance of the manuscript.

### Animal Maintenance and Use

In accordance with institutional policies, mice were housed in a pathogen free vivarium. Pregnant females were identified by the presence of a vaginal plug and the embryos were designated as stage E0.5. When experimentally indicated, animals were fed doxycycline-containing mouse chow (Testdiet, 0.0625% Doxycycline) instead of the normal stock diet. Embryos were dissected from the uterine horn at the indicated stages, fixed in 4% paraformaldehyde, infiltrated with sucrose, and embedded in OCT (Tissue-Tek). The Z/EG line has previously been described [15]. Individual mice harboring the Rx-tTA: TetOp-Cre allele were indentified by PCR using either the primers p1-5′-GGTGCGCCTGCTGGAAGATG and p2-5′-CCATTCCTGCCGTCCGGAACA (95°C, 30 sec; 55°C, 30 sec; 72°C, 30 sec; × 30cycles) to yield a ∼550 bp (pre-flp) or ∼245 bp (post-flp) product or p3-5′-ATGCCCAAGAAGAAGAGGAAGGT and p4-5′-GAAATCACTGCGTTCGAACGCTAGA (95°C, 30 sec; 56°C, 30 sec; 72°C, 30 sec; × 27cycles) to yield a ∼447 bp product.

### Immunofluorescent Staining

Embryonic cryosections were placed on glass slides, rinsed with PBST, blocked in 4% powdered milk, and incubated for 1 hour with a 1∶1000 dilution of phalloidin (Invitrogen, A12381) and Hoechst (Sigma, B-2261) to visualize F-actin and nuclei, respectively.
